# The Influence of Infectious Disease Cues on Purchase Intention for Environmentally Friendly Products

**DOI:** 10.3390/ijerph18168421

**Published:** 2021-08-09

**Authors:** Xingyuan Wang, Yaming Wang, Yi Wang

**Affiliations:** School of Management, Shandong University, Jinan 250100, China; wangxingyuan@sdu.edu.cn (X.W.); 201820348@mail.sdu.edu.cn (Y.W.)

**Keywords:** infectious disease cues, environmentally friendly products, perceived uncertainty, need to belong

## Abstract

Consumers often come across cues of infectious disease in daily life, such as diners coughing in restaurants, commuters sneezing on the bus, or recent news reports about the spread of infectious diseases. In this study, four experiments were conducted to explore the role of infectious disease cues on consumers’ purchase intention for environmentally friendly products (eco-friendly products), as well as the moderating effects of consumers’ sense of power and anti-disease intervention. According to the results, infectious disease cues enhance consumers’ intent to purchase eco-friendly products, and perceived uncertainty and need to belong played a chain-mediated role in the relationship between infectious disease cues and this purchase intention. Consumers’ sense of power moderated the relationship between infectious disease cues and purchase intention. The purchase intention of consumers with a low sense of power (vs. high sense of power) was significantly enhanced when the infectious disease cues were highlighted. Anti-disease interventions also have a moderating effect on the relationship between infectious disease cues and purchase intention. When anti-disease intervention (such as wearing an anti-bacterial mask against airborne diseases) was adopted, consumers’ willingness to purchase eco-friendly products decreased.

## 1. Introduction

Imagine driving to the supermarket and listening to a report on your car radio about the outbreak of an infectious disease in a city. When you arrive at your destination, you park your car and go to the supermarket to buy your daily necessities. Which appeals to you more: environmentally friendly (eco-friendly products) or non-environmentally friendly products (non-eco-friendly products)? Consumers often encounter cues for infectious diseases during daily life. Surprisingly, despite their ubiquity, the impact of contagion cues on consumer behavior has not been fully studied. Such questions have become particularly important as COVID-19 has rampaged around the world.

When an epidemic breaks out, information related to the epidemic is always transmitted through multiple channels and forms, and people pay close attention to it. During the COVID-19 pandemic, stores ran out of hand sanitizers, disinfectants, face masks, shelf-safe food, and even toilet paper. The pandemic has shown that infectious diseases can provoke strong emotional responses that influence consumer choices. In the few studies that have explored how cues of contagion affect consumer behavior, researchers have proposed that such cues trigger consumers’ fear perception. Huang et al. believe that infectious disease cues stimulate the fear of infection followed by the motivation to avoid people, and eventually increase the relative purchase intention of atypical products [1]. Galoni et al. note that when a person is faced with the threat of infectious disease, the main emotional reaction is disgust and fear, which increases the intention to purchase familiar products and even increase the overall purchase quantity [2]. The consensus in the existing research mentioned is that infectious disease cues can trigger consumer fear, which is an emotional response to uncertainty and a lack of personal control over the outcome [2]. However, only when consumers think a situation is serious does fear occur. Exposure to infectious cues does not necessarily produce the fear that the previous studies mention. However, when consumers encounter such cues, they might first have the perception of uncertainty—that is, they are not sure whether they may be infected with the disease, what consequences they will face if they are infected, and how to deal with the disease. Therefore, it is necessary to study the effects of consumers’ perception of uncertainty.

The uncertainty–identity theory (UIT) suggests that when in a state of uncertainty, individuals understand which group they identify with and belong to through self-categorization and deindividuation, and this ultimately reduces the sense of uncertainty [3,4]. The individual’s need to belong is enhanced and he or she will feel a strong desire for social acceptance. To feel more accepted by society, people often constrain their behavior according to the norms and values recognized by others [5], and sometimes even sacrifice their personal interests [6]. A review of the literature shows that people regard objects as self-extensive, a part of themselves [7]: products have a symbolic value to highlight an individual’s identity [8,9]. For example, when individuals suffer from social exclusion, they may repair themselves through luxury consumption [10]. Consuming eco-friendly products is a pro-social behavior because it is good for the environment, altruistic, and morally relevant [11]. Consumers’ environmentally friendly behavior can help them make a good impression on others [11]. Therefore, we believe that infectious disease cues will arouse people’s perception of uncertainty, which will lead to a need for a sense of belonging, and thus increase people’s purchase intention toward eco-friendly products. To the best of our knowledge, this has not yet been considered by current research. Even in the context of the study of fear, there appears to be no research on the relationship between fear and consumers’ purchase intention toward eco-friendly products.

This paper also suggests that when consumers are exposed to infectious disease cues, not all consumers are more likely to purchase eco-friendly products (vs. non-eco-friendly products). The influencing factors can be divided into internal ones and external ones. The individual characteristics play a vital role in our study because consumers with a high sense of power will feel lower uncertainty and have less need to belong when compared with those with a low sense of power after coming into contact with the infectious disease cues. At the same time, anti-disease intervention is a kind of external cause, which has been proved to be an important method to prevent the spread of infectious disease. When effective measures are taken to prevent infection, the consumer will not feel great uncertainty and, therefore, have less need to belong. Therefore, this paper considers the moderating effects of consumer sense of power and anti-disease intervention from the perspectives of individual differences in consumers and the impact of the external factor. Consumers with a different sense of power have different purchase intentions toward eco-friendly products after being exposed to infectious disease cues. Similarly, the presence or absence of anti-disease intervention can significantly affect consumers’ choices. This paper examines the mediating role of perceived uncertainty and need for belonging on infectious disease cues and purchase intention for eco-friendly products, as well as the moderating role of consumers’ sense of power and anti-disease intervention.

In the course of examining these questions, we contribute to existing research in several ways. First, the relationship between infectious disease cues and consumers’ purchase intention was explored. Second, UIT was introduced into the field of consumer behavior and, based on this theory, the mechanism of infectious disease cues influencing consumers’ purchase intention toward eco-friendly products is analyzed. Third, the moderating variables of the influence of infectious disease cues on purchase intention—that is, consumers’ sense of power and anti-disease intervention—were explored, and the boundary conditions of this relationship were given.

## 2. Literature Review and Hypotheses

### 2.1. Infectious Disease Cues

In interpersonal relationships, infectious cues trigger a series of instinctive responses by activating safety-seeking motivations. For example, infectious disease cues increase a preference for symmetrical faces because they are evolutionarily associated with better physical health [12], and these cues increase visual attention to asymmetrical faces because they are associated with disease [13]. This effect can be observed in a variety of other interpersonal processes, including stigma and prejudice [14], social influences [15], and moral judgments [16].

Infectious disease cues can also influence consumer behavior by stimulating the consumer’s behavioral immune system and eliciting emotions. Studies have shown that fear of catching a disease can reduce consumers’ willingness to buy second-hand goods and increase the value of new products [17]. Based on the behavioral immune system, Huang et al. discussed how cues related to infectious diseases can stimulate consumers’ fear of infection, thus increasing the motivation to avoid people and the relative preference for atypical products [1]. When a person is faced with the threat of infectious disease, the main emotional response is disgust and fear, which increases the willingness to buy familiar products and even increases the overall number of products consumers buy [2]. In previous studies, scholars have found that cues can trigger consumers’ fear, which in turn affects consumers’ purchase of second-hand goods, atypical products, and familiar products.

### 2.2. Eco-Friendly Products

Eco-friendly products, or ecologically friendly products, refer to a class of products whose production, use, or treatment process can significantly reduce harm to the environment [18]. Because eco-friendly products can contribute to the improvement of the environment, buying them is also called green consumption [19]. Scholars generally equate eco-friendly products with green ones [20]; although they share the same connotation, differences remain between the two: green products have a more abstract concept and broader connotation, while eco-friendly products have more specific attributes and more specific connotations. From the perspective of product attributes, both eco-friendly products and green products have egoistic (healthier and better personal image) and altruistic (beneficial to the environment) connotations, but eco-friendly products have more prominent altruistic attributes, while green products have no clear direction toward altruistic and egoistic attributes [21]. This study focuses on eco-friendly products with obvious altruistic properties.

Existing studies have explored the influence of information frameworks [21], information demand [22], information appeal [23], product display mode [24], and individual and social factors [25] on purchase intention for eco-friendly products. The negative information frame is more effective than the positive one in promoting green consumption behavior [21]. The demand for descriptive normative information will stimulate the self-interested motive of social belonging needs/social norms, and promote consumers to choose eco-friendly products [22]. The form of the appeal in advertisements (concrete or abstract communication) can also influence purchase intention by influencing consumers’ psychological distance [23]. Through the positive metaphorical correlation between morality and status, Wang et al. explained that the up-and-down display of eco-friendly products affects purchase intention [24]. Mazar found that, in addition to personal factors such as identity, status, and lifestyle, factors at the level of social norms such as ethics, ethical values, and beliefs also stimulated consumers to purchase eco-friendly products [25]. Although there are abundant studies on green consumption, a large number of studies focused on general marketing strategies and consumer characteristics, and there is a lack of research on the impact of external health threats such as infectious disease cues on consumers’ purchase intention toward eco-friendly products from the perspective of external threats.

### 2.3. Infectious Disease Cues and Eco-Friendly Products

Previous studies have shown that infectious disease cues can elicit fear [2], which is an emotional response to uncertainty and a lack of personal control over outcomes [26]. When consumers encounter infectious disease cues, there is a degree of uncertainty about whether they are likely to be infected with the disease and whether the disease poses a threat to their health. When individuals are faced with external threats, they often feel a strong need to belong, because individual power is limited and people seek each other’s support to better cope with threats, have a better chance of surviving, and pass on their genes [27,28]. When the sense of belonging needs to be strengthened to be better accepted by society, people often constrain their behavior in accordance with norms and values recognized by others [5], and sometimes even sacrifice their personal interests [6].

Eco-friendly products are beneficial by improving the environment, so the behavior of buying eco-friendly products is also called green consumption [19]. Green consumption can be associated with the social symbolism of altruism [28], enabling consumers to be more positively evaluated by others [29]. Compared to the consumption of non-eco-friendly products, consumption of eco-friendly products is good for the environment, which can appear to be moral, altruistic, and pro-social [11]. Consumers’ environmental behavior can thus help them form a good impression in the eyes of others [30] and receive affirmation from others [11]. In the case of prominent infectious disease cues, consumers perceive greater uncertainty, generate a greater need for belonging, and then engage in more pro-social behaviors, so they may be more inclined to buy eco-friendly products. Based on this, H1 is proposed.

**Hypothesis** **1** **(H1).** *Compared with non-infectious disease cues, infectious disease cues significantly enhance consumers’ purchase intention toward eco-friendly products.*

### 2.4. Mediating Effect of Perceived Uncertainty and the Need to Belong

Hogg has argued that there are a variety of sources of uncertainty, which means that people are not certain of themselves, their own identity and the environment [3]. This uncertainty is disgusting and uncomfortable [3], which causes emotional and cognitive reactions [31] and makes it hard for people to forecast and plan behavior. People are thus always trying to reduce, control, or prevent uncertainty from happening [32]. Indeed, reducing uncertainty is one of the basic psychological motivations among human beings [2], and it is an important factor driving individuals to display certain behaviors. People seek each other’s support to cope effectively with the great uncertainty generated by external threats and to improve their chances of survival [28,29]. Studies have found that, in the process of human evolution, possible threats such as diseases, dangers, and natural disasters have strengthened the mutual needs of human beings. For example, Schachter found that people prefer to wait with others rather than wait alone when they are about to receive electric shocks [29]. Previous research has shown that the perception of uncertainty can trigger the need to belong.

The need to belong refers to the internal drive to participate in a certain organization or to be attached to a certain group, and to form and maintain stable and positive interpersonal relationships with others [33,34]. UIT also suggests that when individuals feel uncertain, they understand which group they identify with and belong to through self-classification and deindividuation, predicting the attitudes and behaviors of others, which finally reduces the sense of uncertainty [3,4]. In the context of this study, consumers exposed to infectious disease cues are uncertain about whether they will contract the disease, the consequences of doing so, and how to deal with the disease. The perception of uncertainty triggers consumers’ need for belonging. To satisfy this need, the immediate response is to reconnect through active contact with others, or to conform to group norms to be accepted [35]. Another indirect way of coping is to re-establish a sense of belonging through other non-social goals, such as products [36]. Previous studies have found that individuals who think belonging is important have a stronger preference for social acceptance. A strong need to belong may drive individuals to exhibit cooperative or other pro-social behaviors.

The implementation of pro-social behaviors enables individuals to obtain more positive social- and self-evaluation [37], as well as a more positive emotional experience [38]. To some extent, pro-social behaviors can meet an individual’s basic need to belong, such as establishing a connection with others, obtaining a sense of security, and obtaining good psychological feelings [5]. Eco-friendly products are associated with altruism and their consumption is considered a pro-social behavior. Choosing eco-friendly products is thus helpful in enhancing the connection between individuals and others, increasing positive emotions, and meeting the need to belong. Pro-social behaviors can also highlight the moral quality of consumers [11], help individuals to build a good social reputation and gain greater advantages in interpersonal communication or other social contacts [35]. It can thus be inferred that the moral image established by choosing eco-friendly products is conducive to the recognition and acceptance of individuals by groups and organizations and to meet the need to form and maintain stable, positive relationships with others. In the case of prominent infectious disease cues, consumers perceive more uncertainty, which triggers a greater need to belong, which elicits more pro-social behaviors, so they are more inclined to buy eco-friendly products. Based on this, H2 is proposed.

**Hypothesis** **2** **(H2).** *Perceived uncertainty and need to belong play a chain mediating role between infectious disease cues and purchase intention for eco-friendly products.*

### 2.5. The Moderating Effect of Sense of Power

A sense of power refers to a person’s ability to influence others and to have a sense of control over rewards, resource allocation, or punishment; it is a state or tendency of psychological influence [39,40]. Individual sense of power is not only a long-term individual characteristic, but also a kind of immediate psychological state [41] Power brings status, material wealth, respect, and admiration [42], which makes a high-power person think more positively about him- or herself [43], and improves his or her ability to resist threats and fear [44]. Infectious diseases pose a threat to human health and may even lead to social, economic, and informational threats. Becker noted that wealth and status could increase an individual’s ability to cope with unexpected accidents and disasters and change their current weak and helpless situation. Due to the possession of material and psychological resources, high-power individuals have greater psychological security in the face of fear and threats [45]. People with a low sense of power have fewer resources and need to obtain or maintain important social resources through others and achieve their goals by integrating with others [46]. In this study, we believe that, compared with individuals with a low sense of power, individuals with a high sense of power experience greater psychological security and are less afraid of risks when faced with the external threat of infectious diseases. In the context of prominent infectious disease cues, compared with high-power people, low-power people will perceive more uncertainty, resulting in a stronger need to belong, and thus a greater inclination to buy eco-friendly products. Based on this, H3, H3a and H3b is proposed.

**Hypothesis** **3** **(H3).** *Perceived power moderates the relationship between infectious disease cues and purchase intention for eco-friendly products.*

**Hypothesis** **3a** **(H3a).** *Under infectious disease cues, for consumers with high sense of power, their intention to purchase eco-friendly products has not been enhanced significantly.*

**Hypothesis** **3b** **(H3b).** *Under infectious disease cues, for consumers with low sense of power, their intention to purchase eco-friendly products has been enhanced significantly.*

### 2.6. The Moderating Role of Anti-Disease Intervention

In response to the threat of germs, humans have developed a behavioral immune system over time. While the biological immune system acts after bacteria have entered the body in a passive response, the immune system is a preventive immune response designed to prevent bacteria from entering the body in a variety of ways. This can immediately appear in reactive avoidance behavior when smelling something disgusting or seeing another’s pain, stricter sexual attitudes [47], willingness to use a condom [48], avoiding sick people [49], and other avoidance behaviors.

Avoiding disease is now no longer limited to early threat identification and social avoidance: advances in medical technology offer more direct forms of protection. For example, over the past century, public immunization interventions using vaccines have all but eradicated major health threats such as smallpox and polio. Vaccination continues to provide effective interventions against influenza and other infectious diseases [50]. Studies have also shown that public health campaigns to promote hand washing can also help prevent disease [51]. Huang et al. found that actions to fight infection, such as hand washing, can reduce high levels of prejudice against stigmatized individuals due to disease in interpersonal relationship [52]. In view of the effectiveness of these technologies, public health works to prevent the spread of disease, so the threat of infection is eliminated, and the psychological reaction associated with the threat of disease may also arise, while public health interventions to reduce individual fears related to infectious disease cues [1] also reduced uncertainty in individual emotional responses. Studies have shown that public health interventions, such as vaccination, frequent hand washing, and reducing crowds, do reduce consumers’ perceived uncertainty. This study therefore predicts that if significant anti-disease interventions are adopted, the willingness to purchase eco-friendly products, which would be enhanced by infectious disease cues, will be reduced. Based on this, H4 is proposed.

**Hypothesis** **4** **(H4).** *Anti-disease interventions have a moderating effect on the relationship between infectious disease cues and purchase intention for eco-friendly products. Specifically, anti-disease interventions have a weakening effect on this relationship.*

## 3. Study Design and Result Analysis

### 3.1. Study Overview

In this study, four experiments were conducted to evaluate how infectious disease cues affect consumers’ willingness to purchase eco-friendly products and their boundary conditions. In all four studies, we reported on stimulus materials, manipulation, data exclusion, and methods. Study 1 verified that, compared with the neutral control group, consumers were more willing to buy eco-friendly products than non-eco-friendly products in the infectious disease cue situation, which tested H1. In study 2, the external validity of the experiment was enhanced by changing the stimulus. In addition to verifying H1 again, study 2 showed that perceived uncertainty and need to belong played a chain mediating role between infectious disease cues and purchase intention of eco-friendly products—that is, H2 was verified. Studies 3 and 4 verified the moderating effect of consumers’ sense of power and anti-disease intervention, respectively, on the relationship between infectious disease cues and consumers’ purchase intention to eco-friendly products.

Participants were recruited from Questionnaire, a nationwide professional research site similar to Amazon’s Mechanical Turk. All participants were informed that the purpose of the study was to solicit their opinion on eco-friendly products. Next, the participants were asked to read image or textual material and complete the related questions. The scale in this study was adapted from or directly adopted the mature scale. The questionnaire used a seven-point Likert scale, ranging from 1 = Completely disagree to 7 = Completely agree, to rate responses. The scale of perceived uncertainty was adapted from the research of Poortvliet et al. [53] and Kushner et al. [46]. It has three questions: “I am not sure if I will get this disease,” “I am not sure how I should deal with this disease,” and “I am not sure what kind of effects this disease will have on me.” The belongingness needs scale was adapted from that developed by Leary et al. [54], which consists of 10 items, including “I will not be bothered if others reject me,” “I try not to do things that will cause others to reject me or avoid me,” and “I seldom worry about whether others care about me”. The scale of purchase intention was adapted from the research of Dodds et al. [55], including three items: “I may consider purchasing this product”, “I have a high possibility of purchasing this product”, and “I have a high intention of purchasing this product”.

### 3.2. Pretest

**Figure 1:**
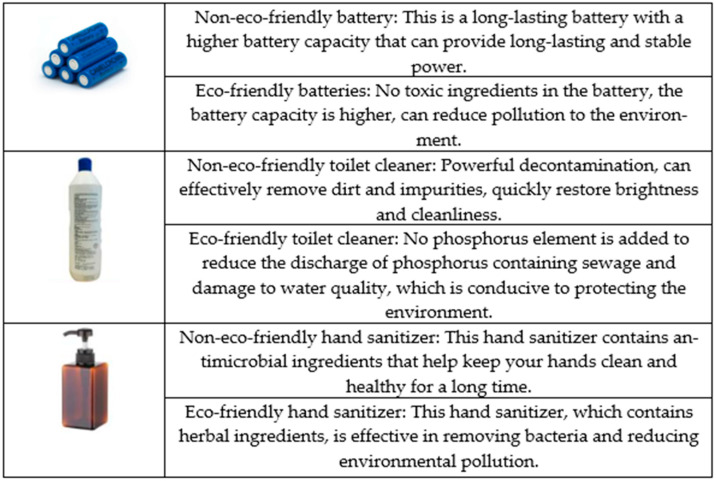
Non-eco-friendly products and Eco-friendly products in the experiments.

Firstly, according to the literature and in-depth interviews with 60 participants, we collected 10 common, real products (bag, laundry detergent, desk lamp, paper, paper cups, battery, plates, stationery, toilet cleaners and hand sanitizer) that are frequently purchased. Each group of products included two types: eco-friendly products and non-eco-friendly products. We then invited experts in the field of marketing to discuss the collected 10 groups of 20 products based on economics and consumer perception of external effects. After the discussion, we chose three groups of products—battery, toilet cleaners and hand sanitizer—as the experimental stimuli, and, according to the information that consumers generally pay attention to when buying products sorted out in the in-depth interview, the experimental content was designed. To control the impact of the brand itself on consumers, the experimental stimuli used virtual brands. To eliminate the impact of product appearance on the purchase intention, the eco-friendly products in one group adopted the same visual design as the non-eco-friendly products. It should be noted that the purpose of selecting hand sanitizers as a stimulant was to test whether hand sanitizers used as a routine disease prevention measure were appropriate for this study scenario. Finally, we obtained three groups, with six products. The experimental stimulants are detailed in Figure 1.

We invited 40 participants (M_age_ = 29.56, female = 64%) to verify the above results, and each product was rated on a 7-point scale (1 = extremely non-eco friendly, 7 = extremely eco-friendly). The results showed that there was a significant difference in the eco-friendliness score between the two groups. The eco-friendliness of the eco-friendly battery (M = 5.50, SD = 0.99) was significantly higher than the non-eco-friendly battery (M = 3.38, SD = 0.92; T = 7.85, *p* < 0.001). The same was true for the eco-friendly toilet cleaners (M = 5.62, SD = 0.94) and non-friendly toilet cleaners (M = 3.00, SD = 0.78; T = 10.73, *p* < 0.001); eco-friendly hand sanitizer (M = 5.50, SD = 0.81) and non-eco-friendly hand sanitizer (M = 3.38, SD = 0.88; T = 8.90, *p* < 0.001).

### 3.3. Study 1

Study 1 examined the effect of exposure to infectious disease cues on consumers’ willingness to purchase eco-friendly products. The manipulation in the infectious disease group was carried out by asking participants to read an article about the transmission of infectious diseases, while the manipulation in the neutral control group was carried out by asking the participants to read an article about the process of organizing their workspace [1]. After reading the manipulation materials, the participants reported their purchase intentions for eco-friendly products or non-eco-friendly products. We predicted that infectious disease cues would increase consumer willingness to purchase eco-friendly products.

Methods: 240 participants were randomly assigned to the infectious disease or neutral control groups. The infectious diseases group was given a chance to read about the three main modes of transmission of infectious diseases: airborne, contact, and fecal/oral. The articles read by the neutral control group described how a student organized his or her workspace in preparation for class. Next, as a test of attention, the participants answered a simple reading comprehension problem about the relevant article (article:infectious disease transmission vs. workspace organization). After viewing pictures and detailed descriptions of batteries (eco-friendly vs. non-eco friendly), the participants were asked about their intention to buy the product. Finally, all participants underwent attention tests, provided demographic information, and reported their buying habits related to eco-friendly products.

**Figure 2:**
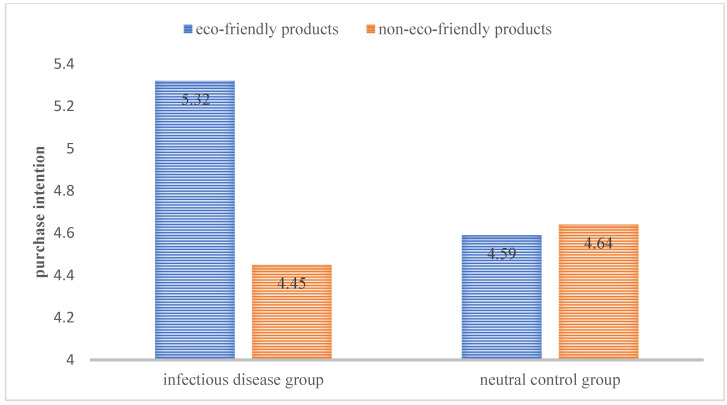
The influence of infectious disease cues on consumers’ purchase intention of eco-friendly and non-eco-friendly products.

Results: 14 participants failed the attention test, so only the results from the remaining 226 participants were analyzed (M_age_ = 31, female = 48.7%); 2 (article: infectious disease transmission vs. workplace organization) by 2 (product type: eco-friendly batteries vs. non-eco-friendly battery) univariate ANOVA showed that there was no significant difference in the daily purchasing habits of eco-friendly products among the four groups (*p* = 0.77). Then, a 2 (article: infectious disease transmission vs. workplace organization) by 2 (product type: eco-friendly battery vs. non-eco-friendly battery) two-way ANOVA showed a significant interaction (F(222) = 4.24, *p* = 0.04). In the eco-product group, the purchase intention among participants with the infectious disease cues (M = 5.32, SD = 1.25) was significantly higher than for the neutral control group (M = 4.59, SD = 1.64; F(1,99) = 6.47, *p* = 0.01). In the non-eco-friendly product group, there was no significant difference in purchase intention between the infectious disease groups (M = 4.45, SD = 1.79) and the neutral control group (M = 4.64, SD = 1.83; F(123) = 0.32, *p* = 0.57), see Figure 2. The experimental results support H1.

Discussion: Consistent with our prediction, the infectious disease cues led to an increase in purchase intention for eco-friendly products compared with the neutral control group. Can a different set of stimuli and manipulating materials confirm the hypothesis? What is the underlying mechanism for this effect? Study 2 selected another stimulus and manipulated materials in a different way to enhance the external validity of the experiment and to verify that consumers’ perception of uncertainty and need to belong play a chain mediating role between infectious disease cues and consumers’ purchase intention for eco-friendly products.

### 3.4. Study 2

In Study 2, we selected stimuli and manipulation materials that differed from those in Study 1 to further verify its conclusions. We also tested the chain mediating effect of perceived uncertainty and need for belonging between infectious disease cues and consumers’ intention to purchase eco-friendly products.

Methods: 95 participants from the questionnaire were randomly assigned to the infectious disease group and the non-infectious disease group. Infectious and non-infectious diseases were manipulated using the same method of previous research [1]. Half of the participants read a piece of news about bird flu, a common infectious disease while the other half read one about heart disease, a common noninfectious disease. Next, after viewing pictures and a detailed introduction of eco-friendly toilet cleaners or non-eco-friendly toilet cleaners, participants were asked about their purchase intention and tested their perception of uncertainty and their need to belong. Finally, all participants underwent attention tests, provided demographic information, and reported their buying habits related to eco-friendly products.

Results: Seven participants failed the attention test, so only the remaining 88 participants were analyzed (M_age_ = 35, female = 47.7%). An independent sample *t*-test was conducted on the daily purchasing habits of eco-friendly products between the two groups. The results showed that there was no significant difference between the infectious disease group (M = 5.00, SD = 1.41) and non-infectious disease group (M = 5.22, SD = 1.46; t = 0.73, *p* = 0.47). The reliability of the uncertainty perception (α = 0.89) and need for belonging (α = 0.97) scales was also good. Univariate ANOVA was conducted for eco-product purchase intention between the infectious disease group and non-infectious group. The results showed that there were significant differences in purchase intention between the infectious disease group (M = 5.80, SD = 1.27) and non-infectious group (M = 4.47, SD = 2.02; F(1,86) = 13.59, *p* < 0.001). Therefore, H1 was supported again, which further verified that the participants exposed to infectious disease cues tend to be more willing to buy eco-friendly products.

To assess the chain mediation of uncertainty and need to belong, ANOVA of perceived uncertainty showed significant differences between the infectious disease and non-infectious groups (F(1,86) = 17.22, *p* < 0.001). The infectious disease group (M = 5.69, SD = 1.10) perceived more uncertainty than the non-infectious group (M = 4.30, SD = 1.92). The need to belong followed a very similar pattern (F(1,86) = 15.02, *p* < 0.001). The infectious disease group (M = 5.72, SD = 1.16) perceived a greater need to belong than the non-infectious group (M = 4.40, SD = 1.95).

To explore the mediating role of perceived uncertainty and need to belong, we conducted a series of mediating analyses (Model 6; Preacher and Hayes, 2004) [56]. There are two categories of independent variable that we encoded. When comparing the infectious disease condition and the non-infectious disease condition, bootstrapping analysis (Hayes and Preacher, 2014) [57] produced a chain-mediated effect through perceived uncertainty and need to belong (β = 0.86, Se = 0.25; 95% CI: 0.39 to 1.39).

Discussion: The results of study 2 support H2 that perceived uncertainty and need to belong play a chain mediating role between infectious disease cue and eco-product purchase intention. Compared with non-infectious disease cues, consumers perceived greater uncertainty under infectious disease cues. Compared with non-infectious disease cues, consumers also perceived a greater need to belong under infectious disease cues. When the need to belong was enhanced, individuals had a stronger purchase intention to gain social acceptance, so they tended to choose more eco-friendly products. At the same time, H1 was tested again in study 2.

### 3.5. Study 3

Study 3 examined the moderating effect of consumers’ sense of power on eco-product purchase intention under infectious disease cues. Based on our hypothesis, we expected that participants with low power would be more willing to purchase eco-friendly products after experiencing an infectious disease cue. The participants with a high sense of power had no significant change in their purchase intention after a similar experience. This study used stimuli and manipulation materials different from those used in Study 1 and 2.

Methods: 160 participants recruited from the questionnaire were randomly assigned to a 2 (slideshow: infectious diseases vs. non-infectious diseases) by 2 (sense of power: high sense of power vs. low sense of power) between-subject experiment.

Sense of power primes. Participants’ sense of power was activated by role assignment and organization chart [58]. The specific operation process was that participants were assigned to one of two roles: leader (high power person) and subordinate (low power person). The high-power person was told that he or she would guide the group members to complete tasks, control the pace of the group activities, and promote the progress of the group activities without being influenced by others. Low-power participants were told that they were under the guidance and control of the group leader, that they needed to report the task completion progress and decision-making results to the group leader in a timely fashion, and that they had to accept the evaluation and assessment of the group leader. A three-layer organizational chart was attached to the right side of the guideline, indicating that the leaders were at the top of the whole structure chart and the subordinates were at the bottom of the whole structure chart. Eighty participants each were primed for low power and high power.

Operational test of sense of power priming. Based on the definition of the sense of power and the existing research, after reading the assigned role, the participants answered the following two questions: “The role assignment makes me feel that I have the ability to guide and influence others” and “The role assignment makes me feel that I have the ability not to be controlled and influenced by others”. A seven-point self-rating was conducted from “strongly disagree” to “strongly agree”.

The manipulation of disease salience used the slideshow of infectious versus noninfectious diseases. The participants were told that they would perform two separate tasks. The manipulation for infectious and non-infectious diseases drew on previous research in which all participants were shown a set of four pictures [1]. The participants were shown a picture of skin and mouth lesions associated with lupus (for example, a rash on the cheek and nose, swelling of the joints). Lupus was chosen to manipulate the participants’ perceptions of contagion because it is a relatively unknown disease. The infectious disease group was manipulated by reading a paragraph in which they were told that the disease was caused by a virus and was highly contagious between people. The non-infectious disease group was told that the disease was genetic, not contagious, and could not be spread from person to person. Next, we asked the participants about their intention to buy the products after viewing the pictures and detailed the introduction of the eco-friendly hand sanitizers. Finally, all participants took attention tests, reported on their usual buying habits for eco-friendly products, and provided demographic information.

**Figure 3:**
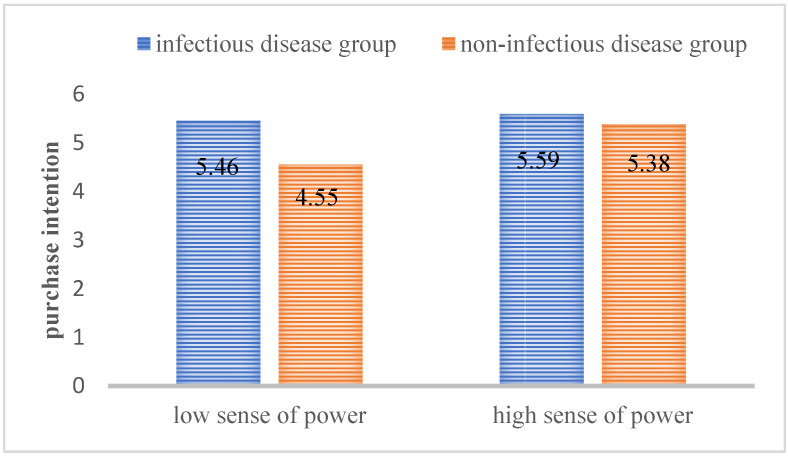
The moderating effect of sense of power on consumers’ purchasing intention of eco-friendly products.

Results: seven participants failed the attention test. Correspondingly, we tested the attention of the remaining 153 participants (M_age_ = 31.46, Female = 56.2%). According to one-way ANOVA of the 2 (slideshow: infectious diseases vs. non-infectious diseases) by 2 (sense of power: low sense of power vs. high sense of power) between-subject design, there was no significant difference in the daily green purchasing habits (*p* = 0.84). An independent sample t-test was conducted on the sense of power between the low sense of power groups and the high sense power of group. The results showed that there was significant difference between them. The score of the high-power primed group (M = 5.68, SD = 0.67) was significantly higher than those of the low-power primed group (M = 2.01, SD = 0.75; *p* < 0.001, t = 31.96), indicating that the priming of power was effective. Two-way ANOVA of the 2 (slideshow: infectious disease vs. non-infectious disease) by 2 (sense of power: low sense of power vs. high sense of power) design showed a significant interaction (F(149) = 4.19, *p* = 0.04). In the case of low power perception, the intention to purchase eco-friendly products under infectious disease cues (M = 5.46, SD = 1.15) was significantly higher than that under non-infectious disease cues (M = 4.55, SD = 1.01; *p* = 0.002). However, as far as the high sense of power was concerned, there was no significant difference in product purchase intention under the infectious disease cues (M = 5.59, SD = 0.87) and the non-infectious disease cues (M = 5.38, SD = 1.17, *p* = 1.00). See Figure 3. Infectious disease cues increase the eco-product purchase intention of consumers with low sense of power, which supports H3, H3a, H3b.

Discussion: Study 3 demonstrated the moderating effect of consumers’ sense of power. The results showed that the participants with a low sense of power were significantly more willing to purchase eco-friendly products when the cues of infectious disease were salient. There was no significant change in the willingness of participants with a high sense of power to purchase eco-friendly products. Compared with consumers with a low sense of power, consumers with a high sense of power experienced a higher sense of psychological security and self-worth, and were less afraid of risks. Compared with those with a low sense of power, those with a high sense of power perceived less uncertainty when experiencing infectious disease cues. This led to less of a feeling of a need to belong. A low need to belong also makes high-power people less interested in purchasing eco-friendly products to meet the need to belong in the context of prominent infectious disease cues. The targeted anti-disease intervention may weaken consumers’ perception of uncertainty, thus reducing their need for attribution and reducing the eco-product purchase intention. Study 4 examined the regulatory effect of anti-disease intervention.

### 3.6. Study 4

Study 4 tested the moderating effect of anti-disease intervention on the purchase intention of eco-friendly products from infectious disease cues. Based on the research hypothesis, we expected that when targeted anti-disease intervention was adopted, the salience of infectious disease cues would not enhance the eco-product purchase intention. In this study, the infectious diseases were described as respiratory diseases. Wearing a mask could significantly reduce the possibility of infection and play a preventive role. As a targeted anti-disease intervention, the influence of infectious disease cues on enhancing consumers’ eco-product purchase intention disappeared.

Methods: A total of 180 participants recruited from the questionnaire were randomly assigned to a 2 (picture: infectious disease vs. non-infectious diseases) by 2 (mask: present vs. absent) within-subject design. The manipulation of infectious disease cues was the same as in study 3. The participants were told that they would perform two separate tasks. As in study 3, all participants were shown a set of four pictures. The infectious disease group was manipulated by reading a passage that told them the disease was highly contagious; the non-infectious disease group was told that the disease was not contagious. Next, for participants in the non-mask group, we let them view the pictures and a detailed introduction to eco-friendly products and then asked them about their purchase intention. Participants in the mask-wearing group viewed a sheet of mask details. It is worth noting that, given the questionnaire setting, this study could not be manipulated by actual mask wearing. To better manipulate the perception of wearing masks, this study was carried out in two steps. First, the mask details page highlighted the mask’s ability to protect against bacteria; the participants were then asked to imagine themselves wearing a mask under the pretext of asking them to evaluate the product. We then let the participants view the picture and a detailed introduction to the eco-friendly hand sanitizer or non-eco-friendly hand sanitizer and asked the participants about their purchase intention. Finally, all participants underwent attention tests, reported on their usual green buying habits, and provided demographic information.

**Figure 4:**
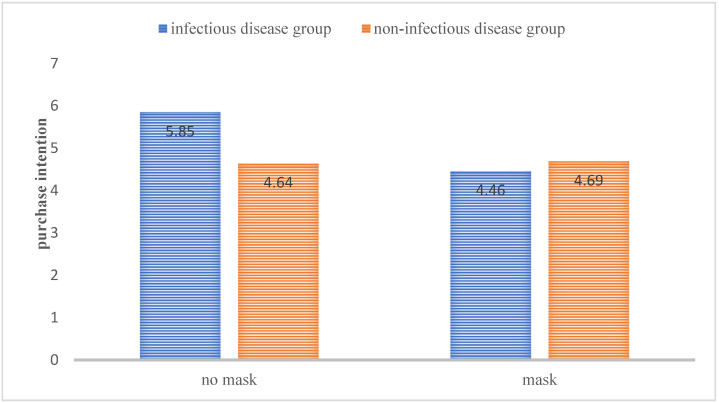
The moderating effect of anti-disease intervention on consumers’ purchase intention of eco-friendly products.

Results: 11 participants failed the attention test. Correspondingly, we tested the attention of the remaining 169 participants (M_age_ = 30, Female = 49.7%). According to one-way ANOVA of the 2 (slideshow: infectious diseases vs. non-infectious diseases) by 2 (Mask: present vs. absent) between-subject design, there was no significant difference in the daily green purchasing habits (*p* = 0.84). Two-way ANOVA of the 2 (slideshow: infectious disease vs. non-infectious disease) by 2 (mask: absent vs. present) design showed a significant interaction (F(165) = 6.77, *p* = 0.01). Without masks, the intention to purchase eco-friendly products under infectious disease cues (M = 5.85, SD = 1.38) was significantly higher than in the non-infectious disease group (M = 4.64, SD = 1.94, *p* = 0.02). However, this effect disappeared when participants assessed and imagined themselves wearing the mask, and there was no significant difference in product purchase intentions between the infectious disease group (M = 4.46, SD = 1.90) and the non-infectious disease group (M = 4.69, SD = 1.86; *p* = 1.00). See Figure 4. Thus, imagining wearing a mask reduced consumers’ willingness to purchase eco-friendly products when infectious diseases are salient, which supports H4.

Discussion: The results of study 4 support H4—that is, after an anti-disease intervention, the salience of infectious disease cues did not significantly enhance consumers’ eco-product purchase intention. When consumers take targeted anti-disease intervention measures, the possibility of infection is reduced, the perceived uncertainty is reduced, the need for belonging caused by the perceived uncertainty is also weakened, and thus the motivation for pro-social behavior is reduced, so consumers are less likely to choose eco-friendly products.

## 4. General Discussion

### 4.1. Theoretical Contribution

This paper makes three main theoretical contributions:

Firstly, the correlation between infectious disease cues and consumers’ eco-product purchase intention was established. This further enriches the research on consumers’ adaptive response to external threats. External threats refer to the actual or potential events that may have a negative impact on consumer well-being, including economic, health, information, environmental, and social threats [59]. Extensive literature in behavioral health [60], the social sciences [61], and the humanities [62] has examined external threats and consequences. In this study, we described the adaptive responses of consumers and the significance of market adaptive responses in the face of infectious disease cues as a health threat.

Secondly, this study improves our understanding of the UIT and is an integration and application of the UIT in the field of consumer behavior. This paper explains the consumption problem of individual commodity choice under the threat of infectious diseases, and theoretically expands and extends the UIT.

Thirdly, we found and tested the mediating effects of uncertainty and need to belong on the relationship between infectious disease cues and eco-product purchase intention. This study introduced and examined the internal effect mechanism of uncertainty and need for belonging on infectious disease cues and eco-product purchase intention, thus providing new and useful information for comprehensive and detailed understanding of the internal decision-making mechanisms for consumers’ green purchasing behavior.

### 4.2. Practical Implications

First of all, given that infectious disease cues will increase the sales of eco-friendly products, marketers should pay keen attention to changes in the external environment, especially external threats, and be prepared to adjust their marketing strategies at any time when promoting and marketing eco-friendly products. For example, marketing strategies for eco-friendly products should consider seasonal patterns of infectious diseases. In general, winter is the peak period of infectious diseases, in which cases are often more serious than they are during the summer and more difficult to control, and consumers are faced with more salient infectious diseases cues. For marketers, understanding this seasonal pattern of infectious diseases is an important basis for promotion decisions for eco-friendly products.

Secondly, this study has shown that people with a low sense of power are more likely to buy eco-friendly products when infectious disease cues are salient. Studies have also found that the sense of power can be generated by situational factors, so enterprises can, through some steps, such as light bass (15 dB) music (studies have proved that light beat music can evoke low power), launch the consumer power state, which to a certain extent, encourage the consumer to purchase eco-friendly products [63].

Finally, this study believes that the need to belong will enhance consumers’ purchase intention for eco-friendly products. When marketing eco-friendly products, marketers should publicize their social value and significance more, so that buying eco-friendly products becomes a way to meet the needs of individual belonging. At the same time, in the face of large-scale external threats (such as COVID-19), relevant government departments should take timely action to strengthen consumers’ need for belonging through positive public opinions, gather people’s support, and urge consumers to pay more attention to group interests rather than personal gains and losses, so as to guide the whole society to buy more eco-friendly products and other pro-social goods.

### 4.3. Research Limitations and Future

In this paper, the influence of infectious cues on eco-product purchase intention was explored, and its internal mechanisms and boundary conditions were analyzed. However, there are still some limitations. Firstly, the infectious disease cues in this study were mainly presented in the form of pictures and text. Although we tried to create more realistic cues for infectious diseases in the experiment, there were still differences from real cues. Secondly, because this was not a field study, although we tried to create a more realistic sense of the shopping presence in the experiment, the way the participants imagined the scene described in the experiment was bound to be different from their feelings in a real shopping scene, which further affected the external validity of the research results. Further verification is needed to determine whether the research conclusions of this paper are equally significant in real shopping scenarios.

This study verified the mediating effects of uncertainty and the need to belong on eco-product purchase intention. Future studies should continue to explore other internal mechanisms of the impact of infectious cues on eco-product purchase intention, such as safety seeking, uncertainty avoidance, and osmosis of control. In addition, some consumer self-traits and characteristics may also influence the perception of uncertainty and the need for belonging, which would then affect the eco-product purchase intention. This could include the self-construction type and the relevant knowledge reserve about infectious diseases. The scale and duration of contagion might also affect consumers’ psychological perceptions. If the scale of infectious diseases rises to a certain level and becomes a national or global epidemic, consumers’ psychological state is bound to change accordingly. Similarly, people are adaptive, and if an epidemic persists for a long time, consumers may respond adaptively. Therefore, future research could further discuss the influence of infectious disease cues on eco-product purchase intention, as well as changes in purchase intention under infectious disease cues.

## 5. Conclusions

Through four studies, we investigated consumers’ eco-product purchase intention, the mediating mechanism of perceived uncertainty and need for belonging, and the moderating effects of consumers’ sense of power and anti-disease intervention under the salience of infectious disease cues. The conclusions can be summarized as follows: for eco-friendly products, consumers’ purchase intention under the salience of infectious disease cues is significantly higher than without such cues. As far as non-eco-friendly products were concerned, there was no significant difference in purchase intention between the infectious disease group and the control group. This indicates that the salience of infectious disease cues significantly enhances consumers’ eco-product purchase intention. We examined the chain mediating effect of consumers’ perceived uncertainty and need for belonging on the relationship between infectious disease cues and eco-product purchase intention. The chain mediating variables of uncertainty perception and need to belong validates the uncertainty–identity theory. This confirms the findings of the uncertainty–identity theory, which suggests that when individuals feel uncertain, they understand which group they identify with and belong to through self-classification and deindividuation, predicting the attitudes and behaviors of others, which finally reduces the sense of uncertainty [3,4]. Compared with non-infectious disease cues, when infectious disease cues were salient, consumers perceived more uncertainty and need for belonging, so they pursued more prosocial behaviors and showed greater eco-product purchase intention, which confirm the consensus in recent studies that when the infectious disease cues are salient, consumers’ purchase intention will be changed [1,2]. In the studies, research confirms that when faced with infectious disease cues, consumers tend to reduce relative preference for typical selection [1] or increases preference for more familiar products [2].

This paper explored the boundary condition of the salience of how infectious disease cues increase consumers’ eco-product purchase intentions through a sense of power. Power brings status and material wealth and improves an individual’s ability to resist threats and fears. People with a high sense of power experience a higher sense of psychological security and self-worth. Therefore, compared with those with low power, those with high power perceived less uncertainty when the infectious disease cues were salient. Power can also increase psychological and interpersonal distance. We also explored another boundary condition: anti-disease intervention. When consumers took targeted anti-disease intervention measures (in this study, infectious diseases were described as respiratory diseases, so wearing masks could significantly reduce the possibility of infection and play a preventive role, and this was targeted in the anti-disease intervention), the effect of infectious disease cue salience on consumers’ eco-product purchase intention disappeared. When consumers take targeted anti-disease intervention measures, their perceived uncertainty decreases because the need for belonging caused by perceived uncertainty is also weakened and the motivation for pro-social behavior is reduced, so they are less likely to choose eco-friendly products.

## Data Availability

Due to the confidentiality of the subjects’ privacy, data can be obtained by contacting the authors.
